# FFF 3D Printing in Electronic Applications: Dielectric and Thermal Properties of Selected Polymers

**DOI:** 10.3390/polym13213702

**Published:** 2021-10-27

**Authors:** David Kalaš, Karel Šíma, Petr Kadlec, Radek Polanský, Radek Soukup, Jan Řeboun, Aleš Hamáček

**Affiliations:** Faculty of Electrical Engineering, University of West Bohemia, Univerzitní 8, 301 00 Pilsen, Czech Republic; karels@fel.zcu.cz (K.Š.); kadlecp6@fel.zcu.cz (P.K.); rpolansk@fel.zcu.cz (R.P.); rsoukup@fel.zcu.cz (R.S.); jreboun@fel.zcu.cz (J.Ř.); hamacek@fel.zcu.cz (A.H.)

**Keywords:** 3D printing, filament materials, dielectric parameters, thermal parameters

## Abstract

The present study is a focused and comprehensive analysis of the dielectric and thermal properties of twenty-four 3D printed polymers suitable for fused filament fabrication (FFF) in electronic applications. The selected polymers include various thermoplastic elastomers, such as thermoplastics based on polycarbonate (PC), polyethylene terephthalate glycol (PETG), and acrylonitrile butadiene styrene (ABS-T). Their overall thermal behavior, including oxidation stability, glass transition, and melting temperature, was explored using simultaneous thermal analysis (STA) and differential scanning calorimetry (DSC). Considering their intended usage in electronic applications, the dielectric strength (*E_p_*) and surface/volume resistivity (*ρ_s_*/*ρ_v_*) were comprehensively tested according to IEC 60243-1 and IEC 62631-3, respectively. The values of the dielectric constant (*ε*’) and loss factor (*ε*”) were also determined by broadband dielectric spectroscopy (BDS). While, on the one hand, exceptional dielectric properties were observed for some thermoplastic elastomers, the materials based on PCs, on the other hand, stood out from the others due to their high oxidation stability and above average dielectric properties. The low-cost materials based on PETG or ABS-T did not achieve thermal properties similar to those of the other tested polymers; nevertheless, considering the very reasonable price of these polymers, the obtained dielectric properties are promising for undemanding electronic applications.

## 1. Introduction

The 3D printing of polymer materials is currently a widely used additive technology, especially in rapid prototyping. Nevertheless, considering the price of injection molding forms, 3D printing is naturally becoming increasingly common in high-mix, low-volume (HMLV) commercial production. A significant advantage of 3D printing is the low price and high availability of 3D printers and the low production costs. Therefore, 3D printing, together with a wide range of commercially available polymer filaments; the possibility of multicolor printing; and the availability of biodegradable and chemically or mechanically resistant materials, is becoming widespread in various production fields, including electronics. On the other hand, the 3D printing of an extensive series of products is time consuming; the final treatment to produce a smooth surface without visible layers is a necessary step; there is limited temperature resistance; and the filaments can absorb moisture and lose adhesion during the printing process [1].

Fused filament fabrication (FFF) can be used in several processes, e.g., for (i) the encapsulation of surface mount devices (SMDs) or printed circuit boards (PCBs) on textile substrates by the thermocompression method [2]; (ii) structural electronics [3,4]; (iii) devices used in explosive atmospheres [5]; (iv) high-frequency electronic applications, in which wireless communication and data transmission are used [6,7]; or (v) power electronics [8]. Despite the fact that in such applications properly designed dielectric and thermal properties are essential for correct function and long-term function reliability, scientists do not address this issue with the intensity that it deserves. 

Previously published papers [9,10] focused on the dielectric properties (permittivity, *E_p_*, or resistivity) of 3D filament materials and presented results for the most common materials, such as, in suitable conditions, biodegradable polylactic acid (PLA) [11], high temperature and impact resistant acrylonitrile butadiene styrene (ABS-T) [12], and chemically resistant polyethylene terephthalate glycol (PETG) [13]. However, various new materials for the use of 3D printing are still being developed. For instance, acrylonitrile styrene acrylate (ASA) is considered an advanced alternative to ABS, possessing better UV stability parameters and prone to less shrinkage than ABS, during the printing process. The 3D filaments based on polycarbonate (PC) are characterized by the properties of high impact and wear resistance and they also possess one of the highest temperature resistances of all polymeric filament materials for 3D printing [12]. Materials based on polyvinyl chloride (PVC) are advanced, when considering their chemical resistance to oils, salts, and water [14]. To date, most studies on 3D printing have focused on the evaluation of mechanical properties [15,16], such as the tensile strength of materials based on ABS [17,18,19,20] and PLA [21,22,23] or their ability to resist pressure [24]. Additionally, the methods to increase their mechanical properties by annealing [25], using an auxiliary heating plate [26], choosing a suitable density and topology of infill [27,28,29], or adding metal particles [30] are examined. Nevertheless, the dielectric properties of a wide range of 3D printing materials produced by different manufacturers have not yet been comprehensively compared. Moreover, information related to the dielectric behavior of a given 3D printing material cannot be found in datasheets or any other recommended materials for electronic applications, in almost all cases.

For the reasons mentioned above, this study addresses the complex characterization of a wide range of 3D filaments for FFF printing technology with intended usage in electronic applications. The dielectric parameters (*E_p_*, relative permittivity, dielectric losses, *ρ_s_*, and *ρ_v_*) were measured since they are crucial to the reliability of many electronic devices, especially in such applications where (i) wireless communication and data transmission are used [31]; (ii) the structures are in direct contact with high-frequency electrical circuits [32]; or (iii) power electronics are the final product [8]. Thermal parameters, such as the glass transition temperature (*T_g_*); the melting temperature (*T_m_*); and the temperature of the first oxidation (*T_ox_*), were also measured because electronic circuits containing 3D printed parts can be exposed to high temperatures or generate heat themselves. Finally, the encapsulation of electronic components or modules, e.g., by employing the thermocompression method [2] on textile substrates using 3D printed objects in the rapidly evolving field of smart textiles [5], can be repeatedly exposed to high temperatures in washing and drying machines. This and other emerging applications of FFF 3D printing for electronics is briefly outlined in the following paragraph.

## 2. Emerging Applications of FFF 3D Printing for Electronics

**Figure 1:**
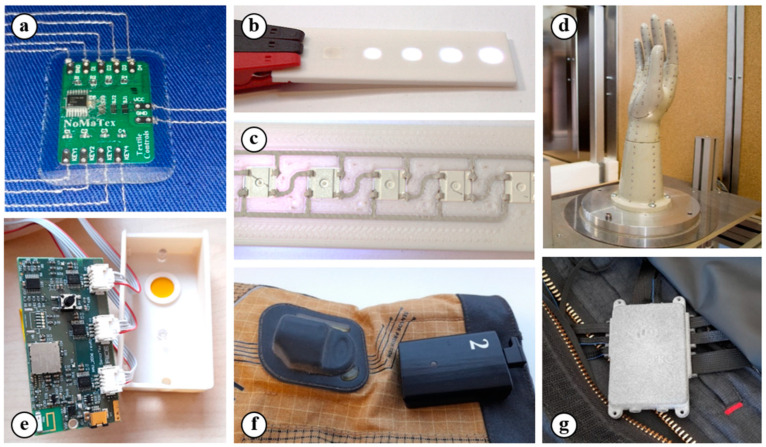
3D printing in electronic applications: (**a**) a PCB and its encapsulation on a textile substrate by a printed polymer component [2]; (**b**,**c**) the front and backside views of a printed polymer component with deposited conductive paths and with an incorporated RGB LED component; (**d**) a printed human hand model with 272 temperature sensors for glove testing [33]; (**e**) the housing for wireless communication electronics; (**f**) a smart firefighter glove with 3D printed housing for the battery [34]; and (**g**) a smart firefighter suit with 3D printed housing for the electronics [5] (the practical results of research activities at the workplace of the authors of the study).

Currently, FFF 3D printing technology is not only used for the rapid prototyping of construction and design elements, it is also suitable for electronic applications (such as smart textiles, structural electronics, high-frequency circuits, and devices for wireless communication) as shown in Figure 1.

### 2.1. Smart Textiles

Smart textiles with electronic components or modules generally require a high chemical, mechanical, and temperature resistance of electrical contacts and their encapsulation. The textiles are exposed to a combination of these influences in washing and drying machines. The study of the possibility of using 3D printing to ensure the connection of conductive structures and their encapsulation using the thermocompression method in smart textile areas is currently in progress [2]. The 3D printed housing with a cavity for the SMD component or PCB is placed on a textile substrate with a conductive pattern and melted into a form using a heat press machine, as shown in Figure 1a. The 3D printed housing provides a pressure force, which creates electrical contact, which is the encapsulation (mechanical and electrical insulating protection) of the component or PCB after it is cooled under a constant pressure.

### 2.2. Structural Electronics

Moreover, 3D printing can be found in structural electronics [3,4], in which the electronic components can be fully embedded into the 3D object. There are cavities for the components and grooves for the conductive tracks on the surface of the 3D printed model in this integration method (Figure 1b,c). the conductive tracks and their contact with the electrical components can be realized by the (i) integration of the conductive wires and foils [35]; (ii) deposition of conductive inks or pastes by aerosol jet printing or dispensing; and (iii) 3D printing of the conductive filaments. On the one hand, the deposition of conductive inks or pastes is suitable for the realization of very fine conductive structures [36,37]; on the other hand, these materials have to be subsequently cured at a high temperature or by a special cure method [38,39]. The deposition of conductive materials can be replaced by the 3D printing of conductive filaments. Fully integrated electronics can be created by a single 3D printer using this method, especially if the currently produced 3D printers support automatic multimaterial printing without lengthy changes to the materials. For conductive tracks, there are available materials filled with carbon or graphene fibers, but their resistivity is too high (up to tens of Ω.cm) [40,41,42]. Such materials are more suitable for creating resistive tracks and resistors and can be used as a primary layer for subsequent electrolytic copper plating [43], as well as for the creation of temperature [44,45] and piezoresistive pressure sensors [46]. The biodegradable composite of polyester and copper with the commercial name Electrifi [41] is practically the only truly electrically conductive material with a resistivity that is less than tens of mΩ.cm. Basic passive components (such as resistors, inductors, and capacitors), antennas used for wireless energy transmission, or high-frequency horn antennas can be created by a suitable topology and a combination of dielectric or resistive materials, together with the conductive material Electrifi.

### 2.3. Devices for Wireless Communication and High-Frequency Circuits

The electrical resistivity and real and imaginary parts of complex relative permittivity are the essential parameters for electronics that work with high frequencies or that are used for wireless communication or charging [47]. The efficiency of charging and the reliability of transmission can be significantly affected by inappropriately chosen materials. In recent studies, a human hand model was printed and developed using polyamide (PA) PA6 filled with short carbon fibers for testing the thermal protection of protective gloves [33]. In such applications, the combination of high mechanical and thermal resistance and good dielectric properties in the used material is required for the reliable function of the testing device. In total, 272 temperature sensors, which generate pulses with widths up to 20 ns, were integrated into this model (Figure 1d). The sensors were mounted on flexible printed circuit boards (PCBs) and integrated into the grooves with cavities for sensors on the inner side of the 3D printed model, which determines that the components were in direct contact with the model [33]. The materials in contact with a PCB can affect its characteristic impedance and cause the deformations and reflections of the transmitted signal. A 3D printed material that is not in direct contact can still affect the function of electronic circuits, especially in devices that use wireless communication. This application is demonstrated in Figure 1e, in which the evaluation board for textile pressure sensors used for measuring edemas with planar antennas for wireless LoRaWAN communication is shown.

### 2.4. Devices with Intended Usage in Explosive Atmospheress

The 3D printed elements can also be used in explosive atmosphere areas and limited by restrictions and special requirements for used materials, e.g., the European standard of equipment intended for use in EXplosive ATmospheres (ATEX) or the International Electrotechnical Commission System for Certification to Standards Relating to Equipment for Use in Explosive Atmospheres (IECEx) system. These standards, in addition to other parameters, observe *ρ_s_*, the ability to transmit an electrical charge from the external atmosphere, and the characteristic temperature parameters [48]. A practical example of such an application can be, e.g., the smart firefighter suit SmartPRO2 containing a 3D printed housing for electronic devices (Figure 1g) capable of measuring the internal and external temperature; monitoring the movement of the firefighter; and integrating LED lighting and a safety belt for the activation of an SOS signal [5]. Smart firefighter gloves complement the suit and further extend its capabilities by measuring the temperature of distant objects (Figure 1f) [34].

## 3. Materials and Methods

### 3.1. Materials

Twenty-three insulating and one dissipative 3D printed polymer were obtained from ten manufacturers and delivered as filaments with a diameter of 1.75 mm. Dissipative materials are intended for applications in which the control of the surface electrical charge is required. The category of dissipative materials is characterized by a *ρ_s_* in the range from 10^4^ to 10^12^ Ω [49]. The extrusion temperatures recommended by the manufacturers (the temperature of the Hotend) for the tested materials vary from 210 to 275 °C, and the bed temperature ranges from 50 to 110 °C (Table 1). Filaments were placed in a drying oven at a temperature of 40 °C, before extrusion, for at least 12 h to minimize the amount of absorbed moisture in the materials. Subsequently, the printed samples were also stored in an oven at a temperature of 40 °C before each measurement. Long-term exposure to humidity degrades the quality of printing, especially for materials based on thermoplastic elastomers (TPEs), thermoplastic polyester elastomers (TPEEs), thermoplastic polyurethane (TPU), and PA or PC [1,50]. A high level of moisture present in the material is indicated by a cracking sound during extrusion, a bad adhesion to the heated bed, an uneven extrusion of the filament from the Hotend, and the presence of voids in printed objects [1]. All these effects deteriorate the mechanical properties of manufactured samples [1,51].

### 3.2. 3D Printing 

**Figure 2:**
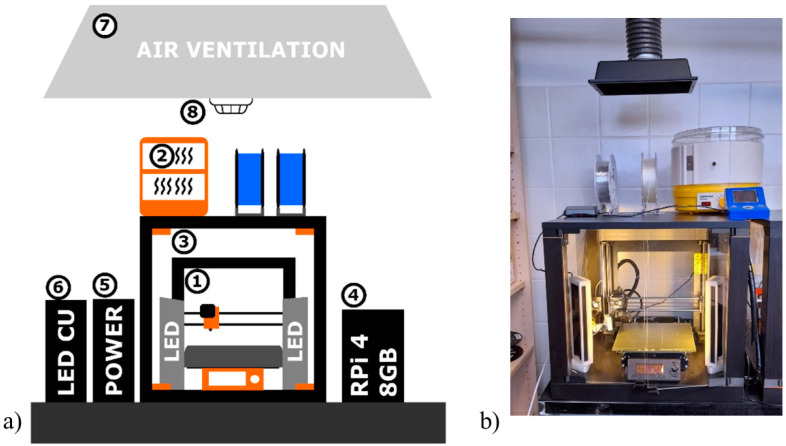
(**a**) Diagram of a custom-built 3D printing workspace: (1) 3D printer; (2) custom filament dryer; (3) custom cover box; (4) RPi 4 for remote system control; (5) external power source; (6) LED control unit (LED CU); (7) air ventilation system; and (8) fire alarm. (**b**) The actual photo of the printer a.

A Prusa MK3S 3D printer (Prusa Research, Prague, Czech Republic) was used for printing the samples. The printer is part of a custom-built workplace allowing for remote control and the administration of the 3D printer (1), as shown in Figure 2. The entire 3D printing workplace was placed on an anti-vibration table to eliminate possible external vibrations, and further consisted of a custom filament dryer (2); a custom cover box (3); a remote control system based on Raspberry Pi 4 (4); an external power source (5); an LED control unit (6); an air ventilation system (7); and a fire alarm (8). The cover box (3) limited the chance of rapid temperature changes and prevented twisting and adhesion loss, especially when printing large objects. An air ventilation system was used to extract harmful and corrosive vapors that formed during the printing process using a Vinyl 303 (PVC) (Fillamentum Manufacturing, Hulin, Czech Republic) filament (sample no. 24 in Table 1).

A flat polyetherimide (PEI) surface was used in the heat bed. When the filament insufficiently adhered to the PEI surface, the special adhesive Magigoo (Thought3D Ltd., Paola, Malta) was used. Thought3D Ltd. produces a series of adhesives with different compositions, each suitable for different filament types. For instance, flexible materials based on TPE and TPU have insufficient adhesion, and the appropriate adhesive is critical for reliable printing. In this study, the parameters of 3D printing were optimized using several steps for individual materials. Magigoo Original Adhesive was used for the Filatech (Al Hamra Island, RAK, UAE) TPEE 40D filament (sample no. 23 in Table 1). Based on the manufacturer data, this adhesive is also suitable for standard materials, such as ABS, ASA, PLA, PETG, and high-impact polystyrene (HIPS). Magigoo Flex Adhesive was used for the TPE 85A filament (sample no. 21) made by Verbatim (Charlotte, NC, USA). Flexible materials made by PM (Chudobin, Czech Republic) (samples no. 20 and 22) and a PP 2330 (sample no. 14) filament were used in combination with Magigoo PP Adhesive, which was also used for filaments based on PC (nos. 8 and 9).

The corrosive vapors formed during the Vinyl 303 (PVC) filament extrusion can also degrade the stainless steel parts of 3D printers. For this reason, a brass nozzle was used to extrude this filament, and the rest of the filaments were nonabrasive materials. Filaments with the addition of abrasives, such as carbon or Kevlar, were extruded by stainless steel nozzles. The diameter of the nozzles was 0.4 mm. The thickness of one printed layer was set to 0.1 mm, whereas the total thickness of the tested samples was 1 mm. The size of the samples for measuring *E_p_* and resistivity was set to 100 mm × 100 mm and 35 mm × 35 mm, respectively, for measuring the permittivity and dielectric losses. All samples were printed with 100% infill.

### 3.3. Simultaneous Thermal Analysis 

Simultaneous thermal analysis (STA) was used to explore the behavior of the tested polymeric materials under thermal stress (controlled heating) up to their complete thermal decomposition. In STA, the temperature dependence of the heat flow was initially evaluated, and weight loss was monitored only as a supplementary characteristic [52]. STA was carried out using an SDT Q600 analyzer (TA Instruments, New Castle, DE, USA) in the temperature range from ambient temperature to 700 °C, with a heating rate of 10 °C/min on samples with a weight of 9.0 ± 0.1 mg. The measurements were performed in an air (oxidative) atmosphere with a 100 mL/min flow rate.

### 3.4. Differential Scanning Calorimetry 

Differential scanning calorimetry (DSC) was used to precisely analyze the critical transient temperatures of the tested samples in the range of 0 °C to 200 °C. The measurements were carried out using a DSC Q2000 analyzer (TA Instruments, New Castle, DEUSA) with a heating rate of 10 °C/min under a nitrogen atmosphere (50 mL/min) and on 9.0 ± 0.1 mg samples. Each material was reheated once during the measurement, whereas the first heating was used for material stabilization and removing any residual moisture. The essential transition temperatures, such as *T_g_* and *T_m_*, which were indicated by the SDT analysis, were recorded more precisely during the second heating via DSC. However, a temperature of 200 °C was insufficient for materials with a high thermal resistance; hence, the entire thermal transition process from a solid (e.g., a glassy or rubbery) to a liquid state (a material with high viscosity) was not possible to record.

### 3.5. Measurement of Dielectric Strength 

*E_p_* (kV·mm^−1^) tests were carried out according to the IEC 60243-1 standard at a power frequency of 50 Hz on samples (100 mm × 100 mm) immersed in mineral oil. The breakdown voltage was measured using a HIGHVOLT testing device (Dresden, Germany) with an LM30 power module, a SM4 control module, and two 110 kV transformers with a 106-kΩ protective resistor. An electrode system consisting of an electrode with a diameter of 25 mm, which was placed in a vessel filled with mineral oil (for surface discharge activity reduction), was selected. Ten breakdown voltage measurements and calculations of dielectric strength were performed for each variant of tested material, and the average values and standard deviations of dielectric strength for all tested materials were determined.

### 3.6. Measurement of Resistivity 

The *ρ_v_* and *ρ_s_* values were determined in accordance with IEC 62631-3 standard under a DC voltage of 1000 V for the *ρ_v_* measurement and of 500 V for the *ρ_s_* measurement. All measurements were performed using an apparatus Keithley (Cleveland, OH, USA) consisting of an electrode system (Keithley 8009), an electrometer with a built-in source (Keithley 6517A), and a computer with control software. Measurements were performed in triplicate for each material, i.e., on three individually prepared samples. The results of the *ρ_v_* and *ρ_s_* measurements are presented as the minimum and maximum values achieved.

### 3.7. Broadband Dielectric Spectroscopy

Dielectric analysis using broadband dielectric spectroscopy (BDS) was primarily used to comprehensively characterize the tested materials regarding their tendency to charge storage and the occurrence of dielectric losses. BDS is based on impedance measurements, and the complex relative permittivity is a key evaluated parameter. More specifically, the values of the real part (dielectric constant, *ε*’) and the imaginary part (loss factor, *ε*”) of the complex relative permittivity for selected frequencies were determined. An Alpha A analyzer (manufactured by Novocontrol Technologies, Montabaur, Germany) with an active ZGS electrode system (cylindrical electrodes with a diameter of 20 mm) was used for testing. The testing voltage was 1 Vrms, and all analyses were performed under laboratory conditions in the frequency range of 0.5 Hz to 1 MHz.

## 4. Results and Discussion

### 4.1. Analysis of Thermal Properties 

As mentioned above, the thermal parameters (*T_g_* and *T_m_*) of 3D printed samples were primarily analyzed using DSC analysis based on measuring the heat flow in the second heating cycle (Table 2). The presented values of *T_g_* are values at the inflection point of the heat flow curve in the area of glass transition [53]. According to [52], *T_m_* was assessed based on the maximum heat flow during endothermic melting. *T_g_* and *T_m_* values depend on the composition of polymers and, for some materials, one of these temperatures was not possible to specify [52,53] because *T_g_* characterizes primarily amorphous materials (e.g., ABS, ASA, and PETG), unlike semicrystalline polymers with a predominant crystalline phase, in which only *T_m_* is presented (e.g., TPE, PA, and PP). Both temperatures were identified for some semicrystalline polymers (e.g., PLA). Increasingly transient temperatures can be detected in polymer materials with more fractions (considerably different molecular masses) or copolymers (e.g., ABS-CF, ABS-AF, ABS ESD, and PC/ABS).

The *T_ox_* value characterizes the temperature stability of materials, and it was measured at temperatures above 200 °C by STA. The beginning of thermooxidation at *T_ox_* was specified based on the heat flow trend as the first unequivocal deflection from the baseline of the thermogram to the positive heat flow values. The determination of *T_ox_* for some materials (e.g., TPE 88A and TPE 85A) was possible; on the other hand, materials, such as TPEE 40D or ASA, were specified by the very gradual beginning of thermooxidation; thus, the determination of the *T_ox_* value was difficult.

STA and DSC confirmed that ASA (material no. 5) is an amorphous polymer with a chemical structure similar to ABS and ABS-T. ABS-T (material no. 1), when compared to ABS, is modified by methyl methacrylate. The glass transition temperatures of the tested materials ASA (103.7 °C) and ABS-T (107.5 °C) were very similar, and the modification of ABS-T by methyl methacrylate had no noticeable influence on the thermograms. The addition of carbon or aramid fibers (material nos. 2 and 3) to ABS should not impact the transition temperatures of the base polymer material. Thus, if two close *T_g_* values of a material were measured, the material has two slightly different structural phases (with different central molecular masses), unlike ABS-T alone. Based on the mass residue measured by STA analysis, the minimal addition (<1%) of carbon or aramid fibers was discovered. The heat flow curve of the ABS ESD (*T_g_* of 109.1 °C) was very similar to that of the ABS-T material (*T_g_* of 107.5 °C), with only one difference; a second, less noticeable transition also occurred at a temperature of 125.8 °C. Moreover, no mass residue by STA at 700 °C was detected in the case of ABS ESD. Because of that fact, the addition of carbon fibers was not confirmed. It is possible to assume that the dissipative property (confirmed by resistivity measurement Table 3) of the material is caused by the modification of the base polymer to the conductive copolymer. The *T_g_* value of neat PC (material no. 9) was detected at 147.5 °C. In contrast, two glass transition temperatures were detected for PC/ABS (material no. 8) corresponding to the *T_g_* of both materials presented, i.e., ABS (117.1 °C) and PC (143.3 °C). cPEST XT and cPEST XT-CF20 (copolyester based on the Eastman Amphora AM1800 3D polymer) had a very significant and similar Tg value (80.9 °C and 81.4 °C). The addition of carbon fibers did not influence the *T_g_* of cPEST XT-CF20 (81.4 °C), unlike ABS materials (material nos. 2 and 3). A different technology of carbon integration can cause this effect because cPEST XT-CF20 contains a relatively large number of carbon fibers (approx. 20% as verified by STA).

The glass transition temperature of materials based on PETG ranged from 78.1 °C to 81.2 °C. The highest *T_g_* was measured for transparent PETG produced by the PM filament, and a lower *T_g_* characterized the pigmented materials (material nos. 11–13). The differences between *T_g_* can be affected by (i) pigments themselves, (ii) different manufacturers and their production technology, or (iii) the quality of raw materials. Nevertheless, the predominant influence of the production process is most likely because of the very similar *T_g_* (78.1 °C and 78.9 °C) obtained for recycled PETG materials with different colors produced by the same manufacturer (EkoMB, Tabor, Czech Republic (material nos. 12 and 13)).

In the case of semicrystalline materials based on PLA, an unquestionable influence of the production process of different manufacturers was observed since the *T_g_* values for material nos. 15–17 were in the narrow range of 59.7 °C to 60.5 °C. However, the difference in their melting temperatures was approx. 15 °C: the *T_m_* of Prusa polymer materials (Prusa Research, Prague, Czech Republic) was 149.6 °C and 150.6 °C, unlike the 164.6 °C of the material produced by Creality (Hong Kong, China). It is likely this difference in *T_m_* values was not only caused by different pigments. All tested PLA materials were characterized by significant exothermic peaks related to their crystallization (maxima at 121.9 °C for PLA no. 15, 126.8 °C for PLA no. 16, and 116.4 °C for PLA no. 17). Moreover, PVC (material no. 24-Vinyl 303) had a *T_g_* value on the thermogram equal to 75.7 °C.

Semicrystalline materials with a predominant crystalline phase, such as PP, PA, and TPE, only had their melting temperature determined. The tested polypropylene material, as a typical semicrystalline thermoplastic material, was characterized by a split endothermal melting peak. The dominant melting temperature (main peak) was recorded as 165.5 °C, and the lower melting temperature of a significantly smaller endothermal peak (corresponding to the minor, less heat resistant fraction) was recorded as approximately 120 °C. The melting temperature of 175 °C recorded for PA 12 (PA12), with the trade name Nylon FX256 produced by Fillamentum, was in accordance with the range observed for PA materials [54]. TPU 98A was characterized by the highest measured melting temperature (195.8 °C) of all the flexible materials tested, which was caused by the melting of rigid chains (block copolymers). In contrast, the melting of soft chains was not observed in the case of TPU 98A or its *T_g_* value because this temperature was below zero, i.e., in a temperature range that was not analyzed in these experiments. In the case of the structurally similar TPEs 88A, 85A, and 32D (material nos. 20–22), a significant difference in the *T_m_* value was recorded between TPE 85A (*T_m_* = 184.5 °C, less significant melting peak); TPE 88A; and TPE 32D (*T_m_* = 155.2 °C and 157.1 °C, respectively, for the most significant melting peak). The same manufacturer produced TPE 88A and TPE 32D, and their results were similar to that of TPEE 40D.

### 4.2. Standardized Tests of Dielectric Properties 

The *E_p_, ρ_v_,* and *ρ_s_* values were measured according to the standards IEC 60243-1 and IEC 62631-3. The obtained results are summarized in Table 3. The *E_p_* was measured ten times, and the average values and standard deviations were determined. The *E_p_* was affected by the quality of the printing for most of the analyzed dielectric properties. The compactness of the structure of the 3D printed object depended on the (i) extrusion of filaments, (ii) the inhomogeneities presented in the object volume (gas bubbles), and (iii) the surface defects. All of these influences can decrease the *E_p_* value. The standard deviation can be used as a scale factor of sample sameness. The *ρ_v_* and *ρ_s_* values were measured in triplicate; thus, the evaluation based on minimal and maximal measured values was chosen.

The *E_p_* values determined for all tested samples ranged from 6.75 kV/mm to 42.25 kV/mm, with one exception: the ABS ESD material. The *E_p_* value of ABS ESD was not measurable because this material is semiconductive. The *E_p_* values for most materials were in the narrow range of 10 kV/mm to 20 kV/mm, whereas those of all the TPEs (material nos. 19–23) significantly exceeded this range. The highest *E_p_* value (32.61 ± 1.53 kV/mm) was measured for PA12 (material no. 18), which is a thermoplastic material. In contrast, materials with carbon fibers (ABS-CF and cPEST-CF) and some PLA materials (material nos. 16 and 17) are characterized by low *E_p_* values, as shown in Table 3. The influence of carbon fibers can be typically demonstrated for the materials cPEST (no. 6) and cPEST-CF (no. 7). While the average value of the *E_p_* of cPEST was 22.73 kV/mm, that of cPEST-CF was only 6.75 kV/mm. Despite the low content of carbon fibers, a significant difference was also measured in the cases of ABS-T (18.37 kV/mm) and ABS-CF (9.32 kV/mm). Hence, the influence of the presence of carbon fibers on the *E_p_* value was observed. Based on these results, it can be determined that materials containing carbon fibers are more suitable for applications in which mechanical strength is required to the detriment of the *E_p_* value. The mechanical strength can also be increased by adding aramid fibers (material no. 3), which have a lower influence on the *E_p_* value than carbon fibers (12.16 kV/mm for ABS with aramid fibers vs. 9.32 kV/mm for ABS with carbon fibers). Interesting differences between ABS-T, PC, and copolymer PC/ABS materials were also discovered based on the *E_p_* results. The *E_p_* value of the PC/ABS material (20.67 kV/mm) was approximately 5 kV/mm higher than that of the PC (material no. 9) and 2 kV/mm higher than that of ABS-T.

**Figure 3:**
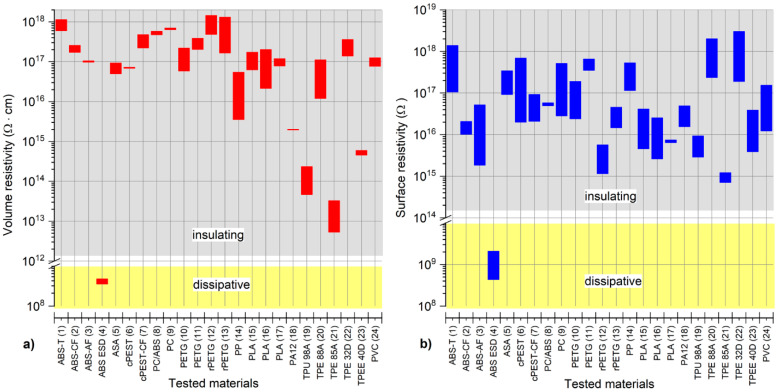
Graphical comparison of the (**a**) volume and (**b**) surface resistivity value ranges for all tested materials.

The measured *ρ_v_* and *ρ_s_* values for all tested materials covered a wide range. Based on the resistivity values, a significant difference from other materials was again observed for the ABS ESD material since this material is more dissipative than electrically insulating materials [49]. Therefore, ABS ESD is useful for applications in whcih electrical charge transmission is required [48]. All other tested materials were electrically insulating materials. A relatively low *ρ_v_* value (less than 1 × 10^15^ Ω.cm) was measured for the flexible materials TPU 98A, TPE 85A, and TPEE40D. Although the *ρ_v_* values for the PA12 and PP materials were typically less than 1 × 10^16^ Ω.cm, the remaining materials were characterized by a *ρ_v_* value of more than 1 × 10^16^ Ω.cm. A significant difference in the range of the *ρ_v_* value for individual materials was also observed. For instance, while the *ρ_v_* values of the ABS-AF material were within a narrow range of 9.76 × 10^16^ Ω.cm to 1.05 × 10^17^ Ω.cm, the PP values were observed in a broad interval range of 3.56 × 10^15^ Ω.cm to 5.43 × 10^16^ Ω.cm. A complex graphical interpretation of the resistivity measurement results obtained for all materials is shown in Figure 3. The distribution of the *ρ_v_* and *ρ_s_* values was similar; however, there was no unequivocal relation between the sequence of the *ρ_v_* and *ρ_s_* values for various materials. Nevertheless, based on the evaluation of the *ρ_v_* and *ρ_s_* values, ABS-T, TPE 32D, TPE 88A, and some PETG materials are very promising for electronic applications.

### 4.3. Broadband Dielectric Spectroscopy 

From all parameters measurable via BDS, the real part (*ε*’) and the imaginary part (*ε*”) of complex relative permittivity were used as the main parameters for this study. Measured *ε*’ and *ε*” values were in the range of 0.5 Hz to 1 MHz, but their values are summarized in Table 4, only for the following critical frequencies: (i) 50 Hz as the frequency of the European power grid; (ii) 140 kHz as the frequency of the most common wireless charging method (inductive coupling) developed by Qi technology [55]; and (iii) 847 kHz representing the subcarrier frequency of the RFID/NFC communication, which is used for data transmission from the passive card to the reader [56,57].

As observed from Table 4, the *ε*’ values at 50 Hz for all tested materials were within the range of 2.1 to 5.6 and did not exceed 3.5 for most materials. The lowest *ε*’ value was measured for PP and TPE 88A. With the increase in frequency, a decreasing trend in the *ε*’ values was observed. An insignificant decline in the *ε*’ value was measured for PP, TPE 88A, and TPE 32D; conversely, a decrease in the *ε*’ value of more than one, with a change in frequency from 50 Hz to 847 kHz, was measured for PA12 and TPU 98A. The measured *ε*’ values corresponded with the theoretical assumptions [58]. However, the observations were, again, very different for ABS-ESD since its *ε*’ value at 50 Hz was 115.58 and was reduced to 53.54 when the frequency changed to 847 kHz. Such high *ε*’ values for ABS-ESD are due to its semiconducting nature, as explained above.

**Figure 4:**
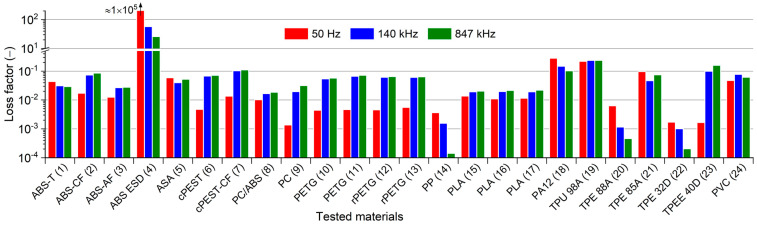
Graphical summarization of the loss factor values measured for all tested materials.

The analysis of *ε*” values as a function of frequency is not as straightforward as in the case of *ε*’ values [59]. With significant differences, the *ε*’’ values were influenced not only by the electrical conductivity of the materials but also by the polarization processes for electrically polar materials. Primarily, the occurrence of polarization processes (dielectric relaxations) causes variations in the *ε*” values between the observed frequencies. The effect of polarization on the measured loss factor was not evident for nonpolar materials, primarily the tested polypropylene materials. Nevertheless, TPE 88A and TPE 32D also presented very similar behavior. In contrast, the polar characteristic is typical for PVC. A slight increase in the PVC *ε*” value at 140 kHz was evident compared to the values at 50 Hz and 847 kHz, indicating a polarization peak. The results of the analysis of *ε*” values further verify the significant similarity of all three tested PLA materials, as well as all four tested PETG-based materials. It should be noted that a minimal difference in the measured *ε*” values for the cPEST materials with and without carbon fibers was observed, whereby a higher increase in *ε*” values due to the addition of carbon fibers was expected. All the differences in the magnitude of the *ε*” value and the differences in its values for individual frequencies are clearly shown in Figure 4.

## 5. Conclusions

When the usage of FFF 3D printing in electronic applications is considered, dielectric parameters are one of the decisive factors that influence the final selection of materials. Although flexible materials have proven exceptional dielectric parameters (*E_p_* > 22 kV/mm), they are not suitable for the construction or self-supporting parts of electronics. Materials based on PCs seem to be universal concerning their high thermal (*T_g_* = 147.5 °C) and mechanical resistivity and above-average dielectric properties (*ε*’*~2.7, E_p_ =* 14.78 kV/mm). Nevertheless, among their disadvantages are the high temperature required for extrusion, high cost, and the need to apply adhesive to the printing bed. 

Low-cost materials based on PETG and ABS-T are characterized by average mechanical, thermal, and dielectric properties; thus, they are suitable for undemanding electronic applications. rPETG has very similar dielectric parameters as PETG and can be considered an interesting material because it is made from recycled raw materials. 

ABS ESD exhibited a noticeably different dielectric behavior and can be categorized as a dissipative material based on its resistivity measurements (*ρ_v_* = 3.58 × 10^8^ Ω.cm and *ρ_s_* = 4.38 × 10^8^ Ω). Hence, ABS ESD can be used as one of the external encapsulation layers for devices in explosive atmospheres, whereby the strict ATEX standard must be satisfied. 

An interesting fact was also discovered for PP, TPE 88A, and TPE 85A materials. The 3D printing process temperatures recommended by manufacturers are on the edge of their thermal stability (*T_ox_*). The Hotend temperature for TPE 32D and PVC materials is also very close to their *T_ox_* values. Furthermore, it was discovered that the thermal and dielectric properties of the same type of polymer, confirmed for TPE (material nos. 20–23) and PLA (material nos. 15–17) materials, which are manufactured by different producers, can slightly differ from each other. Diverse settings of the production process can cause these differences (e.g., the setting of the production line, the chemical composition of raw granular materials, and additives).

It was discovered that a range of filaments is suitable for electronic applications. The injection molding technology can be substituted by 3D printing in the production of HMLV or for rapid prototyping. However, in contrast to injection molding, there are limits to the structural homogeneity of 3D printed objects, which are strongly dependent on the moisture in the filaments and the 3D printer setting, and, as a result, can influence the dielectric properties of polymers.

## Figures and Tables

**Table 1:** Summary of the tested materials and parameters used for manufacturing printed samples.

No.	Material	Color	Manufacturer	Price per 1 kg (USD) ^1^	Print Temperatures (°C) 1st Layer/Subsequent Layers	
					Hotend	Bed
1	ABS-T	Transparent	Filament PM (Chudobin, CZ)	27	255/255	100/110
2	ABS with carbon fibers (ABS-CF)	Black	Kimya (Nantes, FR)	135	260/260	100/100
3	ABS with aramid fibers (ABS-AF)	Black	Kimya	135	260/260	100/100
4	ABS ESD	Black	Smartfil (Alcalá la Real, ES)	70	260/260	100/100
5	ASA	Galaxy black	Prusa polymers (Prusa Research, Prague, CZ)	38	260/260	105/110
6	Copolyester XT (cPEST)	Black	ColorFabb (Belfeld, NL)	63	260/270	90/90
7	Copolyester with carbon fibers XT-CF20 (cPEST-CF)	Black	ColorFabb	79	260/260	90/90
8	PC/ABS	White	Fillamentum (Hulin, CZ)	74	275/275	110/115
9	PC	Natural	Prusa polymers	63	275/275	110/115
10	PETG	Transparent	Filament PM	27	230/240	85/90
11	PETG	Urban gray	Prusa polymers	32	240/250	85/90
12	Recycled PETG (rPETG)	Blue	EkoMB (Tabor, CZ)	20	240/250	85/90
13	Recycled PETG	Milk white	EkoMB	20	240/250	85/90
14	PP 2320 (PP)	White	Fillamentum	84	235/235	60/60
15	PLA	Azure blue	Prusa polymers	27	210/215	60/60
16	PLA	Galaxy black	Prusa polymers	27	210/215	60/60
17	PLA	Red	Creality (Hong Kong, CN)	18	210/210	60/60
18	Nylon FX256 (PA12)	Blue	Fillamentum	82	250/250	90/90
19	TPU 98A	Luminous green	Fillamentum	81	240/240	50/50
20	TPE 88A	Transparent	Filament PM	78	230/230	60/60
21	TPE 85A	White	Verbatim (Charlotte, NC, USA)	99	240/240	50/50
22	TPE 32D	Nature	Filament PM	78	220/220	65/65
23	TPEE 40D	White	Filatech (Al Hamra Island, RAK, UAE)	52	230/230	50/50
24	Vinyl 303 (PVC)	Natural	Fillamentum	66	230/230	80/80

^1^ Price of filaments available in stores in the Czech Republic in 2020, including taxes, customs duty, and import charges.

**Table 2:** Summary of DSC and STA results.

No.	Material	*T_g_* (°C)	*T_m_* (°C)	*T_ox_* (°C)	No.	Material	*T_g_* (°C)	*T_m_* (°C)	*T_ox_* (°C)
1	ABS-T	107.5	-	351	13	rPETG	78.9	-	353
2	ABS-CF	100.2/110.3	-	302	14	PP	-	165.5	239
3	ABS-AF	100.1/111.2	-	336	15	PLA	59.7	150.6	267
4	ABS ESD	109.1/125.8	-	384	16	PLA	60.0	149.6	257
5	ASA	103.7	-	332	17	PLA	60.5	164.6	263
6	cPEST	80.9	-	341	18	PA12	-	178.0	327
7	cPEST-CF	81.4	-	356	19	TPU 98A	-	195.8	322
8	PC/ABS	117.1/143.3	-	337	20	TPE 88A	-	155.2	238
9	PC	147.5	-	324	21	TPE 85A	-	184.5	226
10	PETG	81.2	-	376	22	TPE 32D	-	157.1	249
11	PETG	78.7	-	368	23	TPEE 40D	-	158.8	224
12	rPETG	78.1	-	347	24	PVC	75.7	-	265

**Table 3:** Summary of the dielectric properties of the tested 3D printed materials of DSC and STA results.

No.	Material	*E_p_* (kV/mm)		*ρ_v_* (Ω.cm)		*ρ_s_* (Ω)	
		Average	Standard Deviation	Minimum	Maximum	Minimum	Maximum
1	ABS-T	18.37	4.05	6.02 × 10^17^	1.14 × 10^18^	1.06 × 10^17^	1.38 × 10^18^
2	ABS-CF	9.32	1.59	1.71 × 10^17^	2.57 × 10^17^	1.02 × 10^16^	2.07 × 10^16^
3	ABS-AF	12.16	2.09	9.76 × 10^16^	1.05 × 10^17^	1.84 × 10^15^	5.17 × 10^16^
4	ABS ESD	N/A ^2^	N/A ^2^	3.58 × 10^8^	4.80 × 10^8^	4.38 × 10^8^	2.07 × 10^9^
5	ASA	11.51	2.04	5.03 × 10^16^	9.27 × 10^16^	9.22 × 10^16^	3.42 × 10^17^
6	cPEST	22.73	2.08	6.85 × 10^16^	7.25 × 10^16^	1.99 × 10^16^	6.90 × 10^17^
7	cPEST-CF	6.75	0.67	2.21 × 10^17^	4.79 × 10^17^	2.10 × 10^16^	9.20 × 10^16^
8	PC/ABS	20.67	4.85	4.74 × 10^17^	5.78 × 10^17^	4.94 × 10^16^	5.80 × 10^16^
9	PC	14.78	2.54	6.40 × 10^17^	6.97 × 10^17^	2.82 × 10^16^	5.10 × 10^17^
10	PETG	13.49	4.43	5.89 × 10^16^	2.19 × 10^17^	2.40 × 10^16^	1.89 × 10^17^
11	PETG	15.18	1.66	2.03 × 10^17^	3.86 × 10^17^	3.51 × 10^17^	6.50 × 10^17^
12	rPETG	19.76	2.65	4.85 × 10^17^	1.45 × 10^18^	1.15 × 10^15^	5.64 × 10^15^
13	rPETG	13.91	2.52	1.67 × 10^17^	1.31 × 10^18^	1.46 × 10^16^	4.55 × 10^16^
14	PP	17.68	0.78	3.56 × 10^15^	5.43 × 10^16^	1.15 × 10^17^	5.31 × 10^17^
15	PLA	13.38	1.24	6.25 × 10^16^	1.73 × 10^17^	4.55 × 10^15^	4.12 × 10^16^
16	PLA	7.70	1.26	2.15 × 10^16^	2.01 × 10^17^	2.60 × 10^15^	2.52 × 10^16^
17	PLA	9.61	0.77	7.86 × 10^16^	1.19 × 10^17^	6.40 × 10^15^	7.42 × 10^15^
18	PA12	32.61	1.53	1.96 × 10^15^	2.02 × 10^15^	1.55 × 10^16^	4.90 × 10^16^
19	TPU 98A	23.13	1.50	4.66 × 10^13^	2.34 × 10^14^	2.88 × 10^15^	9.27 × 10^15^
20	TPE 88A	41.52	2.22	1.20 × 10^16^	1.12 × 10^17^	2.36 × 10^17^	2.01 × 10^18^
21	TPE 85A	22.30	1.67	5.34 × 10^12^	3.27 × 10^13^	7.08 × 10^14^	1.21 × 10^15^
22	TPE 32D	36.09	2.17	1.39 × 10^17^	3.60 × 10^17^	1.91 × 10^17^	3.03 × 10^18^
23	TPEE 40D	22.32	1.65	4.57 × 10^14^	5.92 × 10^14^	3.87 × 10^15^	3.88 × 10^16^
24	PVC	22.98	3.76	7.72 × 10^16^	1.25 × 10^17^	1.23 × 10^16^	1.54 × 10^17^

^2^ The dielectric strength (*E_p_*) was not measurable with the standard procedure for other tested materials because this material is semiconductive. During the measurement, the electrical current limit of 15 A was exceeded when the electrical voltage was just 1.5 kV and, hence, the material could not be electrically broken down.

**Table 4:** Summary of the dielectric constant and loss factor values for all tested materials at three selected frequencies.

No.	Material	*ε*’ (-)			*ε*” (-)		
		50 Hz	140 kHz	847 kHz	50 Hz	140 kHz	847 kHz
1	ABS-T	2.65	2.47	2.44	4.38 × 10^−2^	3.10 × 10^−2^	2.91 × 10^−2^
2	ABS-CF	3.00	2.83	2.73	1.71 × 10^−2^	7.32 × 10^−2^	8.51 × 10^−2^
3	ABS-AF	2.61	2.52	2.49	1.25 × 10^−2^	2.67 × 10^−2^	2.75 × 10^−2^
4	ABS ESD	115.58	77.84	53.54	9.92 × 10^4^	5.73 × 10^1^	2.57 × 10^1^
5	ASA	3.22	3.08	3.02	5.90 × 10^−2^	3.98 × 10^−2^	5.23 × 10^−2^
6	cPEST	3.24	3.09	3.00	4.71 × 10^−3^	6.74 × 10^−2^	7.12 × 10^−2^
7	cPEST-CF	4.70	4.45	4.32	1.36 × 10^−2^	1.03 × 10^−1^	1.10 × 10^−1^
8	PC/ABS	2.68	2.62	2.60	1.02 × 10^−2^	1.66 × 10^−2^	1.85 × 10^−2^
9	PC	2.73	2.70	2.67	1.36 × 10^−3^	1.95 × 10^−2^	3.16 × 10^−2^
10	PETG	2.88	2.76	2.69	4.42 × 10^−3^	5.38 × 10^−2^	5.72 × 10^−2^
11	PETG	3.28	3.14	3.05	4.66 × 10^−3^	6.66 × 10^−2^	7.17 × 10^−2^
12	rPETG	3.12	2.99	2.91	4.54 × 10^−3^	6.11 × 10^−2^	6.44 × 10^−2^
13	rPETG	3.10	2.97	2.89	5.55 × 10^−3^	6.10 × 10^−2^	6.36 × 10^−2^
14	PP	2.13	2.12	2.12	3.61 × 10^−3^	1.55 × 10^−3^	1.42 × 10^−4^
15	PLA	2.65	2.57	2.54	1.37 × 10^−2^	1.92 × 10^−2^	2.03 × 10^−2^
16	PLA	2.73	2.66	2.64	1.09 × 10^−2^	1.96 × 10^−2^	2.13 × 10^−2^
17	PLA	2.76	2.69	2.66	1.15 × 10^−2^	1.92 × 10^−2^	2.18 × 10^−2^
18	PA12	4.27	3.05	2.90	2.82 × 10^−1^	1.48 × 10^−1^	1.02 × 10^−1^
19	TPU 98A	5.54	4.38	4.10	2.19 × 10^−1^	2.36 × 10^−1^	2.37 × 10^−1^
20	TPE 88A	2.22	2.20	2.20	6.31 × 10^−3^	1.15 × 10^−3^	4.58 × 10^−4^
21	TPE 85A	4.82	4.65	4.58	9.62 × 10^−2^	4.69 × 10^−2^	7.46 × 10^−2^
22	TPE 32D	2.49	2.48	2.48	1.70 × 10^−3^	1.01 × 10^−3^	2.03 × 10^−4^
23	TPEE 40D	4.38	4.18	4.04	1.65 × 10^−3^	9.92 × 10^−2^	1.58 × 10^−1^
24	PVC	3.35	2.98	2.90	4.75 × 10^−2^	7.72 × 10^−2^	6.13 × 10^−2^

## Data Availability

All data are in the paper.
